# Correction: Intragenomic conflicts with plasmids and chromosomal mobile genetic elements drive the evolution of natural transformation within species

**DOI:** 10.1371/journal.pbio.3003628

**Published:** 2026-01-30

**Authors:** Fanny Mazzamurro, Jason Baby Chirakadavil, Isabelle Durieux, Ludovic Poiré, Julie Plantade, Christophe Ginevra, Sophie Jarraud, Gottfried Wilharm, Xavier Charpentier, Eduardo P. C. Rocha

Supporting Information file S8 Data contains incorrect accession numbers. With this correction, the correct accession numbers are now provided. Please view the correct S8 Data below.

## Supporting information

S8 Data Collection of Legionella pneumophila and Acinetobacter baumannii strains and their measures of transformation rates. (XLSX)
