# Supplementary material for: Intragenomic conflicts with plasmids and chromosomal mobile genetic elements drive the evolution of natural transformation within species
Source: PLoS Biol. 2024 Oct 14;22(10):e3002814. doi: 10.1371/journal.pbio.3002814 (PMC11472951; doi:10.1371/journal.pbio.3002814)
Supplement: S2 Fig — Distribution of the log10-transformed transformation rates in Acinetobacter baumannii (left) and Legionella pneumophila (right) non-transformable (NT) and transformable (T) control strains. (DOCX) [file pbio.3002814.s031.docx]

**S2** **Fig Distribution of the log10-transformed transformation rates in Acinetobacter baumannii (left) and Legionella pneumophila (right) non-transformable (NT) and transformable (T) control strains.** The data underlying this figure can be found in S2 Data.
