# Supplementary material for: Intragenomic conflicts with plasmids and chromosomal mobile genetic elements drive the evolution of natural transformation within species
Source: PLoS Biol. 2024 Oct 14;22(10):e3002814. doi: 10.1371/journal.pbio.3002814 (PMC11472951; doi:10.1371/journal.pbio.3002814)
Supplement: S3 Fig — Absolute variation of transformation rates between 2 strains depending on their patristic distances in Acinetobacter baumannii (left) and Legionella pneumophila (right). (DOCX) [file pbio.3002814.s032.docx]

**S3 Fig Absolute variation of transformation rates between two strains depending on their patristic distances in Acinetobacter baumanni (left) and Legionella pneumophila (right).** Regressions between abs(∆TF) and patristic distance were performed with a linear model (purple line) and with a generalized additive model (GAM default parameters; blue line). The data underlying this figure can be found in S16 Data and the regressions in S17 Data.
