# Supplementary material for: Intragenomic conflicts with plasmids and chromosomal mobile genetic elements drive the evolution of natural transformation within species
Source: PLoS Biol. 2024 Oct 14;22(10):e3002814. doi: 10.1371/journal.pbio.3002814 (PMC11472951; doi:10.1371/journal.pbio.3002814)
Supplement: S4 Fig — Variation of strain transformation rate depending on its patristic distance with the donor strain in Acinetobacter baumannii (left) and Legionella pneumophila (right). (DOCX) [file pbio.3002814.s033.docx]

**S4 Fig Variation of strain transformation rate depending on its patristic distance with the donor strain in Acinetobacter baumannii (left) and Legionella pneumophila (right).** The data underlying this figure can be found in S18 Data.
