# Supplementary material for: Intragenomic conflicts with plasmids and chromosomal mobile genetic elements drive the evolution of natural transformation within species
Source: PLoS Biol. 2024 Oct 14;22(10):e3002814. doi: 10.1371/journal.pbio.3002814 (PMC11472951; doi:10.1371/journal.pbio.3002814)
Supplement: S5 Fig — Log10-transformed transformation rates depending on the percentage of identity shared between the homology arms of the strain and the plasmid donor strain ones in Acinetobacter baumannii (left) and Legionella pneumophila (right). (DOCX) [file pbio.3002814.s034.docx]

**S5 Fig Log10-transformed transformation rates depending on the percentage of identity shared between the homology arms of the strain and the plasmid donor strain ones in Acinetobacter baumannii (left) and Legionella pneumophila (right).** The red dotted line corresponds to the threshold separating transformable from non-transformable strains. The data underlying this figure can be found in S19 Data.
