# Supplementary material for: Intragenomic conflicts with plasmids and chromosomal mobile genetic elements drive the evolution of natural transformation within species
Source: PLoS Biol. 2024 Oct 14;22(10):e3002814. doi: 10.1371/journal.pbio.3002814 (PMC11472951; doi:10.1371/journal.pbio.3002814)
Supplement: S6 Fig — (DOCX) [file pbio.3002814.s035.docx]

**S6 Fig Schematic representation of the models of diverse evolutionary dynamics fitted to the transformation rates (adapted from Landis et al. [34]).** Each colored paths corresponds to the potential evolution through time of the transformation rate of a strain.
