# Supplementary material for: Intragenomic conflicts with plasmids and chromosomal mobile genetic elements drive the evolution of natural transformation within species
Source: PLoS Biol. 2024 Oct 14;22(10):e3002814. doi: 10.1371/journal.pbio.3002814 (PMC11472951; doi:10.1371/journal.pbio.3002814)
Supplement: S7 Fig — (DOCX) [file pbio.3002814.s036.docx]

**S7 Fig Difference between transformable and non-transformable strains of their squared correlation (r^2^) between bi-allelic values at two loci in windows of 500 nt along their genomes**A. Distribution of ∆r^2^ in the 500 nt screened windows in Acinetobacter baumannii (left) and Legionella pneumophila (right). ∆r^2^ was calculated as r^2^_mean_(NT)-r^2^_mean_(T)
B. Distribution of ∆r^2^ along the reference genome divided into 500 nt windows in Acinetobacter baumannii (left) and Legionella pneumophila (right): AB5075 for Ab, Paris for Lp. The windows in which the distribution of r^2^ between transformable and non-transformable populations was significantly different according to a paired Wilcoxon test were colored in green, otherwise they were grey. The highest peaks of ∆r^2^ were annotated with the genes the corresponding window was overlapping. HP stands for hypothetical proteins.
The data underlying this figure can be found in S10 Data.
