# Supplementary material for: Intragenomic conflicts with plasmids and chromosomal mobile genetic elements drive the evolution of natural transformation within species
Source: PLoS Biol. 2024 Oct 14;22(10):e3002814. doi: 10.1371/journal.pbio.3002814 (PMC11472951; doi:10.1371/journal.pbio.3002814)
Supplement: S8 Fig — (DOCX) [file pbio.3002814.s037.docx]

**S8 Fig Distribution of pseudogenized comM and the nature of its interruption if interrupted across the phylogenetic tree of Acinetobacter baumannii depending on the source of the strain**The data underlying this figure can be found in S20 Data.
