# Supplementary material for: Intragenomic conflicts with plasmids and chromosomal mobile genetic elements drive the evolution of natural transformation within species
Source: PLoS Biol. 2024 Oct 14;22(10):e3002814. doi: 10.1371/journal.pbio.3002814 (PMC11472951; doi:10.1371/journal.pbio.3002814)
Supplement: S9 Fig — QQ-plots generated from the outputs of the unitig-based Genome-Wide Association Study on the binary transformation phenotype in Acinetobacter baumannii (left) and Legionella pneumophila (right). (DOCX) [file pbio.3002814.s038.docx]

**S9 Fig QQ-plots generated from the outputs of the unitig-based Genome Wide Association Study on the binary transformation phenotype in Acinetobacter baumannii (left) and Legionella pneumophila (right).** The data underlying this figure can be found in S21 Data.
