# Supplementary material for: Intragenomic conflicts with plasmids and chromosomal mobile genetic elements drive the evolution of natural transformation within species
Source: PLoS Biol. 2024 Oct 14;22(10):e3002814. doi: 10.1371/journal.pbio.3002814 (PMC11472951; doi:10.1371/journal.pbio.3002814)
Supplement: S10 Fig — Alignment of the structure of the DarA_N domain-containing protein in A. baumannii ACICU (A0A4Y3J949 UniProt) (yellow) with the structure of the most significantly associated protein (pangenome family 6781) with non-transformability carried by prophages (blue) in Acinetobacter baumannii. (DOCX) [file pbio.3002814.s039.docx]

**S10 Fig Alignment of the structure of the DarA_N domain-containing protein in A. baumannii ACICU (A0A4Y3J949 UniProt) (yellow) with the structure of the most significantly associated protein (pangenome family 6781) with non-transformability carried by prophages (blue) in Acinetobacter baumannii**
