# Supplementary material for: Intragenomic conflicts with plasmids and chromosomal mobile genetic elements drive the evolution of natural transformation within species
Source: PLoS Biol. 2024 Oct 14;22(10):e3002814. doi: 10.1371/journal.pbio.3002814 (PMC11472951; doi:10.1371/journal.pbio.3002814)
Supplement: S11 Fig — (DOCX) [file pbio.3002814.s040.docx]

**S11 Fig Relatedness of Legionella pneumophila Refseq plasmids and presence of the sncRNA rocRp in their genomes.** This matrix of genomic similarity was obtained with complete linkage clustering based on euclidean distance of their wGRR measure (Material and Methods Plasmid identification in draft assemblies). The data underlying this figure can be found in S22 Data.
