# Supplementary material for: Intragenomic conflicts with plasmids and chromosomal mobile genetic elements drive the evolution of natural transformation within species
Source: PLoS Biol. 2024 Oct 14;22(10):e3002814. doi: 10.1371/journal.pbio.3002814 (PMC11472951; doi:10.1371/journal.pbio.3002814)
Supplement: S12 Fig — (DOCX) [file pbio.3002814.s041.docx]

**S12 Fig Volcano plots showing average effect sizes and significance of the association of the gene families with the transformation phenotype according to** $\boldsymbol{GWAS}_{\boldsymbol{U}}^{\boldsymbol{bin}}$**-cov in Legionella pneumophila and plasmid association with the phenotype.** Each circle stands for a gene family. The size of the circle depends on the number of unitigs that mapped the gene in all the samples. The value on the x-axis corresponds to the average effect size of all the unitigs mapping the gene. The y-axis indicates how significant this effect can be by representing the maximal -log10-transformed p-value adjusted for population structure of all the unitigs of this gene. Significantly associated gene families are above the Benjamini-Hochberg (BH) threshold (red dashed line).
A. Gene families were colored by their frequencies in the collection.
B. Gene families that were carried by a plasmid were colored in green.
The data underlying this figure can be found in S6c Data.
