# Supplementary material for: Intragenomic conflicts with plasmids and chromosomal mobile genetic elements drive the evolution of natural transformation within species
Source: PLoS Biol. 2024 Oct 14;22(10):e3002814. doi: 10.1371/journal.pbio.3002814 (PMC11472951; doi:10.1371/journal.pbio.3002814)
Supplement: S13 Fig — (DOCX) [file pbio.3002814.s042.docx]

**S13 Fig Linear relationship between the number of transformants and the luminescence measured in A. nosocomialis M2 strain**. CFU: colony forming units; RLU: relative luminescence unit. The data underlying this figure can be found in S23 Data.
