# Supplementary material for: Intragenomic conflicts with plasmids and chromosomal mobile genetic elements drive the evolution of natural transformation within species
Source: PLoS Biol. 2024 Oct 14;22(10):e3002814. doi: 10.1371/journal.pbio.3002814 (PMC11472951; doi:10.1371/journal.pbio.3002814)
Supplement: S14 Fig — (DOCX) [file pbio.3002814.s043.docx]

**S14 Fig Benchmarking the luminescence assay by comparing the transformation frequencies determined by selection with a genetic marker and by luminescence values (RLU) in A. nosocomialis M2 strain.** The regression between transformation frequency and RLU was performed with a linear model (blue line). The data underlying this figure can be found in S24 Data.
