# Supplementary material for: Intragenomic conflicts with plasmids and chromosomal mobile genetic elements drive the evolution of natural transformation within species
Source: PLoS Biol. 2024 Oct 14;22(10):e3002814. doi: 10.1371/journal.pbio.3002814 (PMC11472951; doi:10.1371/journal.pbio.3002814)
Supplement: S15 Fig — (DOCX) [file pbio.3002814.s044.docx]

**S15 Fig Variations of transformation rates measured by the luminescence assay in well-documented strains**. Transformation rates were measured for A. nosocomialis strain M2, A. nosocomialis strain M2 ΔcomM (deletion of the gene encoding the ComM helicase is expected to reduce the transformation frequency of heterologous DNA by ~100-fold, see PMID: 29722872), A. nosocomialis strain M2 ΔcomEC (deletion of comEC should result in no transformation = detection limit), A. baumannii strain Ab5075, a well-known transformable clinical isolate, A. baumannii strain Ab5075 ΔabaR (comM repaired since Ab5075 is naturally deficient for comM due to its interruption by AbaR), 2 well-known transformable isolates, A. baumannii A118 and A. baumannii AYE and 2 well-known non-transformable isolates, A. baumannii ATCC17978 and A. baumannii ATCC19606. The data underlying this figure can be found in S25 Data.
