# Supplementary material for: Intragenomic conflicts with plasmids and chromosomal mobile genetic elements drive the evolution of natural transformation within species
Source: PLoS Biol. 2024 Oct 14;22(10):e3002814. doi: 10.1371/journal.pbio.3002814 (PMC11472951; doi:10.1371/journal.pbio.3002814)
Supplement: S16 Fig — (DOCX) [file pbio.3002814.s045.docx]

**S16 Fig Reproducibility of the luminescence assay across replicates in Acinetobacter baumannii and Legionella pneumophila.** The regression between log-transformed RLU of 2 replicates was performed with a linear model (blue line). The data underlying this figure can be found in S8 Data.
