# Supplementary material for: Intragenomic conflicts with plasmids and chromosomal mobile genetic elements drive the evolution of natural transformation within species
Source: PLoS Biol. 2024 Oct 14;22(10):e3002814. doi: 10.1371/journal.pbio.3002814 (PMC11472951; doi:10.1371/journal.pbio.3002814)
Supplement: S17 Fig — (DOCX) [file pbio.3002814.s046.docx]

**

**S17 Fig Comparison of the different phylogenies inferred in *Acinetobacter baumannii* based on different evolutionary features.** Tree_REF_NORECOMB_ is the tree inferred without taking into account recombination on the alignment of concatenated persistent genes ordered according to a complete reference genome (AB5075 genome). Tree_REF_RECOMB_ is the tree inferred taking into account recombination on the alignment of concatenated persistent genes ordered according to a complete reference genome (AB5075 genome). Tree_RANDOM_NORECOMB_ is the tree inferred on the alignment of concatenated persistent genes randomly ordered and that takes into account recombination.
A. Comparison of different evolutionary features in regards to one another and to the transformation phenotype: topological distance (weighted Robinson-Foulds distance wRF), average distance root to tip (dist_root-tip_), phylogenetic signal of the transformation phenotype, fit of the trait to the Brownian motion (BM) and Jump Node (JN) evolutionary models.
B. Pairwise comparison of the terminal branch lengths when provided with an ordered alignment of concatenated persistent genes or a random one.
C. Pairwise comparison of the recombination rate Gubbins estimated when provided with an ordered alignment of concatenated persistent genes or a random one.
D. Pairwise comparison of the cumulated lengths of recombination tracts identified by Gubbins when provided with an ordered alignment of concatenated persistent genes or a random one.
The data underlying S17B Fig can be found in S26 Data, S17C Fig S27 Data and S17D Fig S28 Data.
