# Supplementary material for: Intragenomic conflicts with plasmids and chromosomal mobile genetic elements drive the evolution of natural transformation within species
Source: PLoS Biol. 2024 Oct 14;22(10):e3002814. doi: 10.1371/journal.pbio.3002814 (PMC11472951; doi:10.1371/journal.pbio.3002814)
Supplement: Attachment — Submitted filename: response_comments_review_PLOS_v8.docx [file pbio.3002814.s048.docx]

Reviewer #1:

We thank the reviewer for the work, appreciation and comments on our study.

Assessment:

This manuscript by Mazzamurro et al is an excellent study of the evolutionary benefit of transformation, which combines modelling of genomic data with high-throughput experimental work. It builds on a related previous analysis of Legionella pneumophila by some of the same authors (https://www.ncbi.nlm.nih.gov/pmc/articles/PMC6744872/__;!!JFdNOqOXpB6UZW0!vMjfkIYOFGnj2ZeHHu-cxeyzyRs1HksTNS5hAjeNiQKEVc9D5xw5K3jUl7war8Zv7hT52Vsk9o6nDyP4CMOOAgZ5pA$ ). I have only a few comments that I believe should be addressed in a revised version.

Comments:

1.1: The current analysis of recombination rates using genomic data violates the assumptions of the algorithms being used. The Gubbins analysis is run on a species-wide tree using persistent genes "concatenated in a random order". However, the creators of Gubbins describe such an input as being "fundamentally incompatible" with the software. This is because Gubbins relies on the spatial arrangement of polymorphisms in the genome, which is not accurately represented by a permutation of the persistent gene alignments. Instead, the authors should analyze their data with an algorithm such as fastGEAR.

We answer here to comment 1.1. and 2.1, which are related. While it is true that Gubbins was not originally made to be used for analysis at the species level, it is commonly used to do such analyses. The problem is that Gubbins is not very good at identifying tracts deep in the phylogenetic tree. FastGear was done to search for such tracts. Yet, here we only analyze recombination tracts that are very recent (occurring in the terminal branches) for which replacing Gubbins by FastGear is not of obvious utility. Under these conditions the FastGear paper (Mostowy, MBE, 2017, page 1170 second column) states explicitly that “We see that the false positive rate stayed very low with all methods [incl. FastGear and Gubbins]. Furthermore, overall, fastGEAR had similar sensitivity to detect recent and intermediate recombinations to the other methods, and no method was systematically the best.” We used Gubbins because we are more used to it and because the FastGear paper itself states that for the analysis of recombination at terminal branches the methods are similar.

Since two reviewers suggested that we use FastGear, we retrieved the program and tried to use it to analyze our genomes. After several weeks running in the Institut Pasteur high-performance computer cluster without obtaining an output, we decided to contact the first author of FastGear (Rafal Mostowy, one of our collaborators for other projects). He answered back stating that FastGear was inapplicable to the analysis of such large sets of genomes. Hence, we could not follow the reviewer's recommendation.

Regarding the comment about using a permutation of the persistent genes, we must emphasize that while an initial preprint of this work used such a method, the finally submitted version of the manuscript did not. All of the main results were based on the phylogenetic tree inferred from the alignment of persistent genes ordered according to the reference genome. The additional analyses using either a tree not corrected for recombination or a tree where recombination was inferred in the permutation mentioned by the reviewer presented in Figure S17 was to demonstrate that whether or not we consider recombination (accurate or not) for the inference of the phylogenetic tree, it does not modify the conclusions of the different indicators we used to characterize the transformation trait nor does it change the evolutionary patterns of the trait. This shows the strength of our conclusions on the evolution of the trait regarding the analysis of recombination, since three different methods provide qualitatively similar results.

**Action:** We have now mentioned the caveats regarding Gubbins and clarified the issue of the permutations in a dedicated paragraph in the Methods section.

1.2: How much variation in transformation rate is explained by the different mobile genetic elements, and how much by defence systems such as restriction enzymes? Restriction enzymes can have a strong inhibitory effect on the acquisition of insertions by transformation (e.g. <https://www.ncbi.nlm.nih.gov/pmc/articles/PMC3573125/__;!!JFdNOqOXpB6UZW0!vMjfkIYOFGnj2ZeHHu-cxeyzyRs1HksTNS5hAjeNiQKEVc9D5xw5K3jUl7war8Zv7hT52Vsk9o6nDyP4CMOr_LxYEA$> ), and therefore might strongly affect the results of the assay of transformation rates. The authors should quantify how susceptible their assay is to the effects of restriction by comparing the rates of acquisition of nanoluc in a mutant lacking a restriction system strongly associated with transformation rates, relative to rates in the parental strain.

We agree with the reviewer that R-M can be a barrier to transfer and thus influence the rates of transformation. This was recently demonstrated on *A. baumannii*, one of the model species tested (<https://www.ncbi.nlm.nih.gov/pmc/articles/PMC10287943/>, cited in the text). The R-M system discovered in this study restricts transformation of replicating plasmid but has a lesser effect on transformation of DNA recombining with the chromosome (which is what is tested in our assay). Even so, the transforming DNA used in our assay is extracted from an *E. coli* strain whose methylation system (dam) protects from this *A. baumannii* R-M. We quantified the influence of the R-M systems on transformation rates in our manuscript in the section “MGE shape transformation rate variations”. In response to this comment, we now quantified how much variation in transformation rates could be explained by mobile genetic elements and by restriction modification systems. We performed a phylogenetic logistic regression expressing the binary transformation phenotype as a function of the number of mobile genetic elements inhibiting transformation (phages in Ab and conjugative systems in Lp) and of the number of restriction modification system a strain carries. As expected, both were significatively and negatively associated with transformation but phages in Ab and conjugative systems in Lp had a stronger inhibitory effect than restriction modification systems (Ab: Phages: effect=-0.97, p=4.3x10^-3^; RM: effect=-0.61, p=3.55x10^-8^ and Lp: Conjugative systems: effect=-0.76, p=1.4x10^-4^; RM: effect=-0.41, p=1.09x10^-9^).

**Action**: The new statistical analysis is now included in the manuscript and a mention on the production of methylated DNA was added to the methods.

1.3: The discussion of why the RocRp RNA is found on plasmids is interesting. Three possible explanations that might be considered are: (a) These plasmids might be episomal in Lp or another host species, such that the RNA would be beneficial when they were integrated - do they carry an integrase? In the absence of an integrase, do they have a dif-like sequence (https://www.biorxiv.org/content/10.1101/2021.09.12.459815v1.full__;!!JFdNOqOXpB6UZW0!vMjfkIYOFGnj2ZeHHu-cxeyzyRs1HksTNS5hAjeNiQKEVc9D5xw5K3jUl7war8Zv7hT52Vsk9o6nDyP4CMPwTdMiNg$ ) that can exploit host recombinase activity to insert into the genome?

These are large conjugative plasmids, so they are not expected to integrate at the dif site using the Xer system. Actually, we could not find a single integration of these plasmids in hundreds of genomes of the species. It is true that they might be integrative in other species, but this does not satisfactorily explain why RocRp is never found in integrative elements in Lp when it is so often found in plasmids. Into the bacterial chromosome we looked for the presence of an integrase in their genomes. We searched for tyrosine (PF00589) and serine recombinases (PF00239 and PF07508) with *hmmsearch* (*hmmer/3.3.2*). No tyrosine recombinase nor serine recombinase were found. To test whether these plasmids would have the ability to be integrated using a dif site we searched for homologous regions to the 29-nucleotide conserved sequence of the Lp dif site (Nicholson et al, *mBio*, 2022) in rocRp-carrying plasmids and found no hits. We also searched for XerL homologs in rocRp-carrying plasmids and found no hits. These plasmids do not present any dif/Xer system that could help them integrate into the genome. Putting all this together, we find very unlikely that these plasmids frequently integrate the chromosome.

**Action**: We have detailed these analyses in the text.

(b)     The plasmids are highly variable in gene content (e.g. <https://www.nature.com/articles/s41598-022-09721-9__;!!JFdNOqOXpB6UZW0!vMjfkIYOFGnj2ZeHHu-cxeyzyRs1HksTNS5hAjeNiQKEVc9D5xw5K3jUl7war8Zv7hT52Vsk9o6nDyP4CMMbUCLH1Q$> ) - the RocRp genes may be protecting themselves from being deleted from the plasmids by homologous recombination

This is an interesting hypothesis: recombination between plasmid core genes could result in deletion of RocRp, which would be protecting itself. To test it we estimated the relatedness of all Refseq Lp plasmids with the wGRR measure (Material and Methods Plasmid identification in draft assemblies). We clustered them by their wGRR (complete method, Euclidean distance) and obtained 11 families of plasmids. The resulting matrix can be found in Figure S11. Among these families one family of 8 plasmids (wGRR >= 0.6) encompassed all the plasmids carrying rocRp (5/8). Plasmids carrying rocRp were all genomically very similar sharing with each other a wGRR always superior to 0.77 suggesting that their plasmid content is very conserved. The presence of rocRp on these plasmids might indeed prevent their gene content from being shuffled by homologous recombination.

**Action:** We have added a sentence in the discussion to open this possibility. We also added Figure S11.

(c)     Lp is highly unusual in not being commonly infected by prophage, but still containing many ICEs. Is it possible that plasmids benefit from mobilisation by ICEs or episomal elements that encode conjugative systems that could aid the plasmids' spread? Therefore the plasmids may shut down transformation to preserve the ICEs.

It is not very likely that RocRp plasmids are mobilized by ICEs. The RocRp plasmids carry the set of genes expected for an autonomous conjugative plasmid. The conjugative transfer of one of them (pLPL plasmid) was even shown in Durieux et al, PNAS, 2019. They should not need ICEs to be mobilized between cells. To the best of our knowledge there is no case known of a conjugative plasmid being mobilized by ICEs.

**Action:** We added a sentence suggesting that RocRp would be present to preserve chromosomal MGEs in a relation of positive epistasis with the plasmid.

1.4: The authors identified elevated levels of recombination in the persistent genes flanking MGEs. However, numerous MGEs drive recombination specifically in the flanking DNA, without any role for transformation. In particular, conjugation can drive transfer of nearby DNA (e.g. <https://doi.org/10.1073/pnas.0803654105__;!!JFdNOqOXpB6UZW0!vMjfkIYOFGnj2ZeHHu-cxeyzyRs1HksTNS5hAjeNiQKEVc9D5xw5K3jUl7war8Zv7hT52Vsk9o6nDyP4CMMK5Driwg$> ), and prophage can transfer neighboring sites through specialized or lateral transduction (https://doi.org/10.1371*2Fjournal.ppat.1007878__;JQ!!JFdNOqOXpB6UZW0!vMjfkIYOFGnj2ZeHHu-cxeyzyRs1HksTNS5hAjeNiQKEVc9D5xw5K3jUl7war8Zv7hT52Vsk9o6nDyP4CMN_01D0tA$ ). How do the authors rule out such alternative explanations?

This is a very pertinent observation. We cannot rule out such alternative explanations.

**Action:** we have now indicated this.

Minor comments:

1.5(a) It would be helpful to include the hypothesis that transformation primarily facilitates adaptive evolution more explicitly in the Introduction, as it is mentioned in the Results

We now include this hypothesis among the ones that explain the existence of natural transformation in the introduction.

1.6(b) Fig. 4: it would be helpful to label the polymorphisms associated with the biggest impacts in the volcano-type plots

For the sake of visibility in Figure 4, we only labelled the gene families whose annotation was known and that had either a strong effect size or were very significant. The whole set of gene families that are displayed in figure 4, their p-values, effect size and frequency is listed in Table S6.

**Action:** we have now indicated this in the legend of Figure 4.

1.7(c)     It has recently been suggested that the capsule of A. baumanii affects its transformation rate (https://urldefense.com/v3/__https://www.biorxiv.org/content/10.1101/2024.02.15.580542v1__;!!JFdNOqOXpB6UZW0!vMjfkIYOFGnj2ZeHHu-cxeyzyRs1HksTNS5hAjeNiQKEVc9D5xw5K3jUl7war8Zv7hT52Vsk9o6nDyP4CMOZ07Ck7Q$ ). Did the authors identify any capsule loci in their analysis?

We have tested this following the reviewer’s comment as indeed this could be an important point. We analysed capsules by searching for intact capsule locus in every strain with *Kaptive v.2.0.3*. All strains presented an intact capsule locus. We thus found no evidence that capsule inactivation was causing the emergence of transformable strains. We did not find either any capsule loci as a determinant of transformation in our GWAS analysis. But we cannot exclude the possibility of an effect on gene expression that could not be identified bioinformatically.

**Action**: We now mention this in the section “Transformation rate variations depends on its molecular pathway” and “MGEs shape transformation rate variations” of the results.

Reviewer #2:

We thank the reviewer for the positive and careful assessment of our work.

Assessment:

In their manuscript, Mazzamurro et al. assembled a very interesting dataset to investigate natural transformation and its distribution within bacterial species. They did so at a relatively large scale, with hundreds of isolates being measured for two important bacterial species with different lifestyles: Legionella pneumophila (Lp) and Acinetobacter baumannii (Ab). This very useful dataset is then united with whole genome sequencing of all the isolates to ask questions about the distribution of natural transformation with respect to the phylogeny of the species, the evolution of this trait, the genetic variants associated with variation in this phenotype, and the role of mobile genetic elements. The manuscript is well written and easy to follow, and the underlying data represents, to the best of my knowledge, a unique view on the distribution of natural transformation within bacterial species in terms of the number of isolates being assessed. The "competition" between
transformation and mobile elements (previously proposed on this very journal) is compelling, although difficult to conclusively prove through what is essentially an observational study confirming a previous hypothesis. The authors are however careful to note the limitations of their analyses, leaving careful readers with plenty of information to independently assess the work presented in this study.

I want to focus my critique of this study on two methodological aspects, for which I am more qualified to speak. They would necessarily need to be addressed if this manuscript is to be published in this high visibility journal.

Comments:

2.1 Use of gubbins to detect recombination events within a species: there is an ongoing discussion in the field of microbial genomics as to whether it is appropriate to apply recombination methods based on the "Clonal Frame" model across "strains". By "strain" here I indicate what is also sometimes known as a "lineage", which indicates a set of very closely related set of isolates. This limitation is acknowledged in a publication describing a competing method to detect recombination between (and within) lineages (10.1093/molbev/msx066). Regardless of where the authors stand on this debate I think they should acknowledge that the way they identify recombination may be inappropriate, and thus invalidating for many of their analyses that depend on that. But even better, the authors may try to use fastGEAR to obtain a different list of recombination events to be used to test the robustness of their subsequent analyses.

Please see comment 1.1. which is related.

2.2 The GWAS analysis is using a very lenient corrected p-value threshold, which leads the authors to associate a very high number of gene families to the transformation phenotype. Since they used pyseer on variants encoded by unitigs, they could follow the recommendation to use the number of unique variants presence/absence patterns to derive a more sensible p-value threshold (see: [https://urldefense.com/v3/__https://pyseer.readthedocs.io/en/master/usage.html*number-of-unique-patterns__;Iw!!JFdNOqOXpB6UZW0!vMjfkIYOFGnj2ZeHHu-cxeyzyRs1HksTNS5hAjeNiQKEVc9D5xw5K3jUl7war8Zv7hT52Vsk9o6nDyP4CMPbW5y78g$](https://urldefense.com/v3/__https:/pyseer.readthedocs.io/en/master/usage.html*number-of-unique-patterns__;Iw!!JFdNOqOXpB6UZW0!vMjfkIYOFGnj2ZeHHu-cxeyzyRs1HksTNS5hAjeNiQKEVc9D5xw5K3jUl7war8Zv7hT52Vsk9o6nDyP4CMPbW5y78g$) ). I suspect that the number of significant hits will be significantly reduced, which may bring more clarity to this section. I would then also suggest that the authors generate a QQ-plot to verify that their association analysis is well corrected.

Concerning the correction for multiple testing, we respectfully disagree. The pyseer documentation is not very assertive with respect to the use of Bonferroni. It just states "One way to pick the threshold for significance is to use a Bonferroni correction with the number of unique variants". It is quite common to use BH instead of Bonferroni when the number of tests is high, e.g. Nakagawa, Behavioral Ecology, 2004 (2500 citations). This is because Bonferroni is well known to be excessively conservative when the number of tests is high and especially when they may be correlated (as is the case of testing unitigs from a gene). We agree that the number of significant hits will necessarily be much lower using a Bonferroni correction, but this will not necessarily be the correct result. Of note, a key goal of our GWAS is to study the population of hits among MGEs. For example, changing the type of correction would not significantly change the key conclusions depicted in Figure 4 which relate to the mass of unitigs from MGEs being on one side of the graph.

**Action:** As suggested, we added to the Supplementary Figures (Figure S9) the QQ-plot that showed that the association analysis was well corrected in both species.

Medium point:

2.3- L595: "The threshold between transformable and non-transformable strains was set based on the maximum of the transformation rates of a non-transformable strain". Why using an extreme value as a threshold when the authors have so many replicates for the non-transformable strains? Wouldn't it make more sense to use the mean/median/upper quartile? What if the maximum value happens to come for a replicate that gave a very high reading for technical reasons? Any change in their threshold would of course potentially affect all analyses using the derived binary phenotype, and so more clarity is needed here. Additionally, the GWAS could potentially also be carried out on the continuous phenotype, which would not be affected by this problem, although I recognize that a binary phenotype is more intuitive in this study

There are two points here, one pertaining to the threshold and another to the kind of data.

A) We understand the concern which arises partly because we failed to show enough data on this point. Hence, we now add in the Supplementary tables and Supplementary Figures the distribution of the OD-corrected luminescence values of the non-transformable strains (Table S2, Figure S2): Lens strain in Lp (120 replicates) and engineered ∆*comEC* strain in Ab (28 replicates). This shows clearly that there is no strong effect of outliers in our definition of a threshold. The use of the maximum had the goal of providing a threshold beyond which we do not expect to class a strain as T when it is actually NT. This is now explicit in the text.

**Action**: This is now stated in the text.

B) We agree with the reviewer that in this context the binary phenotype is more intuitive. We are now making analyses with the continuous phenotype to answer to a complementary question: what are the positive determinants of the variations in transformation rates? However, we feel that adding this very extensive analysis to this paper would lengthen excessively the article and lower its focus. We will make it the subject of a future article.

**Action:** None taken

Minor points:

2.4 - L211 "its effect is small and non-significant when phylogeny is not accounted for" <- not sure what this means exactly

We removed the statement since it seems arguable to highlight a test we know is not very correct (because it does not account for phylogenetic structure).

2.5 - L308: "Most of the strongest genetic determinants of transformation inhibition are in MGEs": this is not quantified

We reformulated the sentence (also pinpointed in comments 3.12 and 4.9).

2.6 - Figure 4A: the y-axis label says "log10" but I assume the authors did a natural log transform, as ln(0.05) will yield a value of ~3. This should be corrected

The y-axis here represents the log10-transformed of the p-value adjusted for population structure (lrt-pvalue) but not corrected by Benjamini-Hochberg (BH). The BH threshold (dark red line) of 0.05 is set at the lrt-pvalue that once corrected by BH is equal to 0.05.

**Action:** Information added to the legend.

2.7 - Figure 4B: it seems like most genes in Lp belong to the ICE category, which might indicate that the way they are annotated is too lenient for Lp?

We agree that most pangenome families seem to belong to the ICE category. However, we doubt that it is due to a too lenient definition of ICE for Lp. We used a delimitation of ICEs that is fairly stringent (genes at less than 10kb of a conjugative system). In Proteobacteria ICEs are most often larger than 60kb and in two of the three most frequent types they are on average 80-90 kb (Cury, NAR, 2017). Of note, we did not observe as many pangenome families belonging to ICE in Ab which suggest this difference might lie in the important amount of ICEs carried by Lp when compared to Ab.

**Action:** We now mention the average size of different types of ICEs in the text (Methods).

2.8 - L432: what are the alternative hypotheses that are less simple than the one the authors propose?

The existence of many non-transformable strains could also be the result of a differential expression of the genes involved in the transformation process. Their expression is known to be tightly regulated and changes in their regulation might modify the transformation rates. The presence of a transcription inhibitor of any of these genes might lower the transformation rate of the bacterium and render it non-transformable.

**Action:** The text in discussion was modified to spell this alternative hypothesis more explicitly.

2.9 - L465: I was a bit confused by the last sentence in the paragraph. Could the authors spell this idea out a bit better?

Despite non-transformable strains being counter-selected, we can still observe many strains whose transformation rates are low enough to qualify as non-transformable. This seems a paradox but could actually be explained by the existence of the intragenomic conflict between MGEs and transformation. Indeed, any transformable strain can suddenly acquire MGEs that would render it non-transformable.

Action: we have clarified the sentence

Reviewer #3:

We thank the reviewer for the very thorough review of our text and the numerous comments.

Assessment:

I enjoyed reading this paper. It presents an enormous effort from both an experimental and computational perspective, and the insights the authors reach with this pair of datasets are compelling. In particular, the transformability GWAS is quite a feat and the MGE-related results therein are convincing and impactful. The writing feels a bit convoluted and wordy in places, which could make accessibility to a broader audience more challenging, so conciseness could be improved and redundancies removed to reduce the total length. A detailed model figure could help. Their model makes sense but there are places where cause-and-effect are not established, and so chicken-and-egg situations set themselves up. For example, even if there is a correlation between acquiring MGEs and loss of competence, that doesn't necessarily mean that loss of competence is why those lineages appear prone to extinction. What if it's MGE load causing a fitness problem? Similarly, MGEs do not
have to directly inactivate or suppress natural competence for the model to work, since they might also be more prone to accumulate in lineages that lost competence for other reasons. Thus there is pervasive language that makes assumptions about cause and effect that are not needed or can be relegated to the discussion. I also ask the authors to expand and detail more about their novel luminescence assay for transformation, which allowed them to assay a very large number of strains. While I think that the assay seems reasonable, there is insufficient detail to address potential artifacts that could cause their luminescence measurements to not accurately measure transformability. There are also various places where the comparison between the two species breaks down that require further explanation, along with some other more minor issues.

Comments:

3.1.      The authors need to show validation of their luminescence assay. Only showing a wild type and a transformation null mutant is okay but corroborative transformants/CFU measures as in traditional assays for at least 3-4 genetically distinct isolates with varying intermediate transformation rates, ideally more, is needed (also, can comM be added?). Surely the authors already have these data. There are a variety of potential artifacts that might arise in the context of assaying a diverse set of strains within a species. The authors controlled for differences in biomass by normalizing luminescence to the optical density of the cultures at the time of the assay, but this does not discount expression differences of the Nluc construct related to growth phase, natural variability in the regulation of competence, and several other possible confounders. These issues need not be perfectly addressed but a clearer demonstration that the luminescence quantitatively correlates with
transformation frequency using traditional assays should be shown. Similarly, they state that the measurements are reproducible and did 3-6+ replicates, but no actual spread of LU/RLU measurements across replicates were shown except buried in Table S6, which shows pretty big differences between assay; for some strains, they have up to 120 measurements that were presumably done as controls across assays. Some additional exploration or supplementary information about potential batch effects should be addressed, since otherwise the authors only show this data in Figure 1A. Some of the overall patterns seen (e.g. jump process, etc) could also arise with data that were semi-randomized, so ensuring that these rapid transitions aren't because of non-transformation-related luminescence variability is important.

These are all valid points, and we do have data validating the transformation assay, which are now included in the Supporting Information. These specifically address the following points:

1. Testing the linearity of the assay:

We have measured the luminescence values of serial dilutions of transformed bacteria. We isolated 3 transformants of the *A. nosocomialis* M2 strain. They were grown to obtain cultures whose titers were determined (colony-forming unit, CFU, per 100 µL). Then, ten-fold serial dilutions were made and the luminescence of 100 µL suspension was determined. This results in a plot (Figure S13) in which we can see a linear relationship between CFU count (equivalent to the number of transformants) and luminescence. This is true down to 2x10^2^ CFU, which means that we can detect 20 transformants in a well.

2. Benchmarking the assay by comparing transformation frequencies determined by selection with a genetic marker and luminescence values:

This was done by transforming the *A. nosocomialis* M2 strain with varying amounts of the plasmid (which conveniently carries *Nluc* but also a genetic marker, *kanamycin*). After transformation, the culture was used to determine the transformation frequencies (this is the ratio of the determined CFU count of transformants (on selective plates with kanamycin) and the total CFU count (non-selective plate)). The luminescence of the same culture was measured.

Luminescence measurements and transformation frequencies had a good fit using linear regression (R^2^=0.95)

3. Variations of transformation in selected strains, including, as suggested, testing the effect of *comM* (deleting it in *comM*-proficient strain, and restoring it in a *comM*-deficient strain). We tested the assay with well documented strains, including mutants whose transformation defects were well known.

- *A. nosocomialis* strain M2;

- *A. nosocomialis* strain M2 *ΔcomM* (deletion of the gene encoding the ComM helicase is expected to reduce the transformation frequency of heterologous DNA by ~100-fold, see PMID: 29722872);

- *A. nosocomialis* strain M2 *ΔcomEC* (deletion of *comEC* should result in no transformation = detection limit).

- *A. baumannii* strain Ab5075, a well-known transformable clinical isolate (the positive control in all assays);

- *A. baumannii* strain Ab5075 Δ*abaR* (*comM* repaired). Ab5075 is naturally deficient for *comM* because of AbaR interrupting it. Removing AbaR should restore *comM* and increase transformation rates;

- *A. baumannii* A118. A well-known transformable isolate;

- *A. baumannii* ATCC17978. A well-known non-transformable isolate;

- *A. baumannii* ATCC19606. A well-known non-transformable isolate;

- *A. baumannii* AYE. A well-known transformable isolate.

The results are to be found in Figure S15.

4. Batch effect:

To show the good concordance between replicates and the absence of batch effect, we plotted the distribution of RLU values of each replicate against the first replicate RLU values and provided the adjusted r-square of the corresponding linear regression in both species. The plots are now shown in Figure S16.

**Action:** All the experiments done to validate the luminescence assay were now added to the Material and Methods.

3.2.      The authors obliquely address the issue of whether sequence divergence between the recipient chromosome and the homology arms of their transforming plasmid might drive patterns of luminescence by testing whether related strains had correlated luminescence levels. They report no relationship, but: (A) this isn't directly testing divergence between the relevant loci, and (B) the stats appear to conflict for Ab and Lp, suggesting that these organisms are not comparable in this respect, though they are treated as if they are.

3.2.A.      Divergence between a pair of strains can vary dramatically in sliding windows across a genome, especially given recombination among lineages. Thus, the correct test would be to look at the divergence between the actual homology arms (targeting a minor pilin operon for some reason) of the wild-type lab strains and the recipient strains' orthologous sequences (assuming synteny of these 2 kb arms across strains, but if not, that would also reduce transformation rates). Even identity between donor and recipient still does not show a strong correlation, this still does not exclude variable divergence of donor and recipient as making contributions to the the observed luminescence.

The choice of targeting the *pilMNOP* operon lies in its broad conservation across all strains. Besides in absence of this operon, the cassette could not integrate and the observed phenotype would be non-transformable, which is also what we would expect for a strain lacking the *pilMNOP* operon.

We searched for the homologous arms of the transforming plasmid in the recipient chromosome of each strain of both collections using blastn (-task blastn, blast+/2.10.1). In all strains homology arms had more than 99% identity (covering more than 50% of the query) with the donor plasmid. We measured the correlation between percentage of identity and the transformation rate (measured luminescence. The plots of transformation rates as a function of the percentage of identity between homology arms are to be found in Figure S5. In Ab, transformation rate was not correlated with the divergence observed on the homology arms (rho=-0.026, p=0.54). In Lp, there was a significant correlation between transformation rate and divergence at the homology arms: rho=0.50, p<2.2x10^-16^. This correlation was still valid when reducing this test to the closest strains of the plasmid donor strain (patristic distance <0.04 substitutions per site, rho=0.48, p<2.2x10^-16^). The correlation coefficient is quite important in Lp but the variance is so high that we did not find any form of scaling or normalization that could correct for it. However, transformation rates are known to be robust to sequence divergence of 1-5% (doi: 10.1093/nar/gkv1546; doi.org/10.1111/1462-2920.15342) which is much higher than what we observed here (>99.9%). Thus, the correlation observed in Lp between sequence divergence and transformation rates might be indirect. So, the sequence divergence between the recipient chromosome and the homology arms of their transforming plasmid is not affecting the patterns of transformation rates in Ab but could explain a part of the variation in Lp.

**Action**: This is now stated in the text.

3.2.B.      Stats on at Lines 144-146 and Figure 1A contradict the statement that variance in luminescence does not correlate with phylogenetic distance. This appears true for Ab but not Lp, and the Figure clearly shows that clades of closely related Lp have correlated luminescence values.

We agree that our statements are contradictory. There is a significant association in Lp, but the effect size is low. This is now corrected accordingly (see also 3.2.A).

3.3.      This is not a major criticism, but regarding the transformation assay, I was surprised to find that the authors are using an intact circular plasmid as donor for transformation, rather than releasing the cassette with its homology arms, which should facilitate translocation and recombination. I would expect higher rates of transformation, but perhaps there are details of these assays or these organisms that differ from some other naturally competent organisms.

Indeed, releasing the cassette could have been done. However, this is (1) not needed because the plasmid cannot replicate in the tested species, thus transformants can only be obtained through recombination with the chromosome. And (2) release of the cassette would result in lower transformation frequencies. The initial step of transformation is fragmentation of the transforming DNA, this has higher chance to occurs outside of the cassette when cloned on a plasmid, lowering the chance of cleavage happening within the nanoluc gene or within the homology arms.

**Action:** Information was added in the methods section.

3.4.      The Lp and Ab trees are quite distinct, and I understand how the authors are using that here, however, it is noteworthy that all Lp isolates are clinical (maybe all from the same hospital system?), whereas Ab isolates were a more diverse collection of both environmental and clinical. Could this explain some of the discrepancies seen here, or is this sampling bias a potential confounder in comparing these collections?

It is difficult at this stage to understand if there are links between transformation rates and virulence. There might be indirect associations. For example, in *Acinetobacter* where resistance is frequent in clinical isolates, the resistance is often encoded in mobile genetic elements. This was briefly mentioned before about the ComM inactivations that are more frequent in clinical strains because of AbaR islands which encode antibiotic resistance genes. When testing for association between the source of the strain and the transformation rates, environmental strains were indeed more transformable than clinical ones (Table S11 phyloglm T2). Following this comment (and 3.19), we collected the differences observed between Ab and Lp and used them to build a paragraph in the discussion.

**Action:** A paragraph summarizing these differences was added to discussion.

Other comments:

3.5 Lines 164-178: This is fine but needs a better way to motivate or explain.

The paragraph on the evolution of transformation rates as a jump process was extensively rewritten to take into account this comment and comment 4.2.

3.6 Lines 180-200: Lack of caution about correlation and causation. Later, the demonstration that these lineages have a high load of MGEs gives an alternative explanation.

The prediction is that the loss of transformation leads to accumulation of MGEs because there is antagonism between the two. This fits the data. If we understand correctly, the inverse reasoning would be that strains with more MGEs are less fit. This makes sense and does not need to involve natural transformation. But it does not explain why the strains with many MGEs are less transformable (except if there is antagonism). So, the causal link seems to be the same, the antagonism between transformation and MGEs. If there is no antagonism, then there is no explanation for the accumulation of MGEs in non-transformable strains.

**Action:** We have added a final set of sentences to clarify our thought.

3.7 Lines 208-210: Comforting, but is this not slightly confounded by the bias of non-transformable to be at the tips?

When looking at the recombination rate and their dependence on the transformation phenotype we are only focusing on recombination rates at the tips. Hence, the higher frequency of non-transformable strains at the terminal branches is accounted for.

3.8 Lines 213-217/Figure 2A: This presentation is strange. The "CLRT" here are logs higher than a genome size, so some form of scaling might help these numbers make more sense. It would also be useful to report n within each observation, since it's unclear how many are in each set. But most importantly, the text is noting the discrepancy between Ab and Lp but otherwise ignoring the fact that Lp did not support the stated observation.

Concerning the “CLRT”, the cumulated lengths of recombination tracts had to be scaled by the length of the terminal branches to allow comparisons between groups. Hence the difficulty of adding another form of scaling. We added a small sentence to explain this scaling in Figure 2A caption. We agree with the reviewer that we did not properly underline the discrepancies between Ab and Lp in this intermediate conclusion. This is now corrected. The number of observations was also reported for each group in each species directly on Figure 2A.

3.9 Lines 224-226/Figure 2B: This is a nice demonstration that the non-transformable strains have experienced less recent recombination, but this does not require any allusion to whether this recombination is "adaptive".

We agree. The term “adaptive” has been removed.

3.10 Lines 237-271: Are missing minor pilins known to be required or only sometimes? This section again pretty much ignores Lp. What about gspH, fimT in Lp? Might be worth acknowledging other types of variation could alter transformability, since several of these genes return in the next section.

In this specific part of the text, the focus on *Acinetobacter* was due to the very few genes lacking in Lp. Still, we agree that some genes are lacking in Lp and more frequently in the non-transformable strains. We have now detailed this.

In what concerns the minor pilins, not all of them are essential for transformation. In Lp, transposon-sequencing was used to find the ones that were essential or not: "The Tn-seq data show that a putative pilin PilE (Lpp0681) appears to be required for transformation, while five genes upstream of *pilE* (lpp0686 to lpp0682) and respectively annotated as encoding PilC/PilY1 and minor pilins PilX, PilW, PilV, and GspH/FimT appear to be dispensable." (Hardy et al, Journal of Bacteriology, 2021). Strains lacking *gspH/fimT* in our collection had lower transformation rates than the ones having it (on average 0.8 fold lower). This information is now added in the text.

3.11 Line 293: Can comM be pointed out on Figure 4A?

We added *comM* to the annotated gene families in Figure 4A.

3.12 Line 308: This is a misstatement. MGEs are the most abundant but not "the strongest determinants of transformation inhibition", since there are often other stronger associations. Also, despite recombination among these strains, it is not at all clear that all these MGEs are "determining" the lower transformation frequencies; they are associated. They could be linked by being trapped in a poorly transformable genetic background, rather than directly inhibiting transformation. Some may, but this accumulation of MGEs in these backgrounds does not require that this be so. It could also be that the lack of "chromosome curing" means they're accumulating in these backgrounds without any active inhibition, as sort-of alluded to in the section starting at Line 364.

We have not meant to imply that all MGEs are determining the rates of transformation (not even that they are always the strongest determinant). We corrected the text (see also comment 2.5).

3.13 Line 409: "We found very little association of transformation rates and population structure…" in Ab, but the stats and figure for Lp suggest that at least some clades and groups of closely related strains share similar transformation rates.

We agree. Point related with 3.2B, answered in 3.2B.

3.14 Line 413-430: It is satisfying to see the slight increase in LD breakdown in transformable over non-transformable strains. This is a great observation, even though it is a weak effect. However, this discussion assumes that transformation rates would correlate with recombination rates but transformation often not lead to genetic change, so breakdowns in the correlation could be related to the availability of genetically distinct donors. E.g. competence could be high in situations where most available DNA is from a highly similar cell, and so transformation rates would be high. Another explanation for the only weak correlation is that the benefits of natural competence are only weakly connected with its effect on recombination.

We agree with this criticism and thank for the suggestions that were added to the discussion.

3.15 Line 437: Ref 39 is to a mutant rather than a natural isolate; this might be the wrong citation. The observation with comM is really interesting but this strong affect was only in Ab. I don't think there was any evidence for this working in Lp. Why not? Is the transformation phenotype different for this mutant? The discussion in general seems to somewhat overlook Lp. What is the nature of the comM inactivations in Ab? Are they different MGEs? Are they targeting comM or accumulating there? This and the overall model might require a detailed figure.

The citation was removed.

*comM* is mostly interrupted in Ab by AbaR resistance islands that are derived from Tn7-like elements and were found to be confined to *Acinetobacter* spp (Bi et al*, Antimicrobial agents and chemotherapy*, 2019). These elements target preferentially *comM*. We did not observe any interruption of *comM* in Lp. We thus searched for the Tn7-like AbaR backbone in Lp genomes and found none. The absence of Tn7-like elements does not fully explain why *comM* interruptions are observed in Ab and not in Lp since some comM interruption in Ab are not always caused by Tn7-like elements. In addition to AbaR, *comM* is also interrupted by other IS and defense systems notably in environmental strains. We summarized the distribution of *comM* inactivations across the phylogenetic tree in Figure S8. One possible explanation might lie in the different lifestyles Lp and Ab have. Since Lp is intracellular, the likelihood of recombining with distant kin cells might be lower than for Ab thus rendering *comM* function less critical for solving heterologous recombination and thus natural transformation success.

*comM*-interrupted mutants have a reduced transformation rate as it can be observed in the figure answering comment 3.1.

**Action:** We now detailed the source of *comM* interruption across the Ab phylogenetic tree in the Supplementary Figures and completed the Method section concerning AbaR backbone search.

3.16 Lines 449-453: This represents a completely distinct hypothesis for the evolution and maintenance of the competence machinery that is not listed in Figure S1 or discussed elsewhere but really does represent an important potential mechanism for the maintenance of natural competence: i.e. the machinery may have pleiotropic roles in similar or related processes, or even that interacting with extracellular DNA could have benefits beyond food or sex.

We agree with the reviewer that the maintenance of the competence machinery could be explained by its pleiotropic role. However, since Figure S1 focuses on the direct evolutionary roles of transformation per se and not on the evolution of its machinery for other purposes, we did not add this hypothesis to the figure.

**Action**: We now mentioned this in the introduction.

3.17 Lines 455-467: "Justifies" is the wrong word here. It is worth noting that observations in the Pasteurellaceae (in Ref 41) led Rosie Redfield to entertain the idea of “clade level" selection for competence from her much more limited data. The observations here with MGEs accumulated into non-transformable strains and that these are groups are biased to the tips of the tree.

We agree that the use of the word “justifies” might not be adequate and we modified it accordingly. We had mentioned Redfield’s paper in the context of another reference. But indeed, her evolutionary model of competence with frequent loss of the trait and non-transformable strains being selected against on the long term is consistent with what we also observed.

**Action:** The reference to this model was now included in the discussion.

3.18 Lines 469-483: I don't know that the "chromosome curing" model requires that MGEs block competence in all cases but they should still be able to accumulate in non-transformable backgrounds. This is the chicken-egg thing again. Clarifying the model here is important.

See answer to comment 3.6.

**Action:** we have edited the text for clarity.

3.19 Lines 518-529/Table S6: More information about these isolates is warranted. Are any of the Lp results here affected by the sampling of only clinical isolates? Is there additional metadata to associate with this table, e.g. time frames isolates were collected, etc.

In Lp, only clinical isolates were sampled because it is the only way to get both the actual isolate for experimental quantification and the corresponding genomes. There was no design in the sampling. Random isolates were taken from a collection of Lp sampled across French hospitals from 2018 to 2020. No additional metadata are available. See also comment 3.4.

3.20 Lines 530-536: More details about nLuc and the targeting are warranted. How is nanoLuc expressed and under what control? Plasmids were used straight without cutting?

NLuc is expressed from a synthetic promoter, a *Ptac*. This promoter is normally repressed by *lacI*, but since both tested species lack the lac operon, the promoter is strong and constitutive.

Plasmids are indeed used uncut (see 3.3).

**Action:** This information was now added in methods.

3.21 Lines 538-551: Are the quality filtered assemblies indicated in Table S6 or just excluded?

Assemblies filtered out due to quality were simply excluded.

3.22 Lines 553-586: Were there corroborative measures of transformability using other assays? RLU values reported in Table S6 are highly variable. Was anything done to normalize batches to controls or other?

The reproducibility of the results could be found in Table S7. To better show the good concordance between replicates, we plotted the distribution of RLU values of each replicate against the first replicate RLU values and provided the adjusted r-square of the corresponding linear regression in both species. No normalization was performed.

**Action:** The plots are now shown in Figure S16.

3.23 Lines 588-599: It is reassuring that the luminescence was normalized to OD, but were any checks done to ensure no correlation between OD and RLUs? If highly variable growth rates mean that assays were done with cells in different growth phases, this could affect interpretations substantially.

See answers of 3.1.

3.24 Lines 600-646: This is a good treatment but does remind me that in the GWAS results and discussion, it may be worth bringing up that many of the hits outside MGEs etc could be things that affect the regulation of competence that might be somewhat undetermined.

Point related to comment 2.8 and answered in 2.8.

3.25 Lines 648: Thanks. This is useful. How does this work with the "random gene order" versus "ancestral gene order" versions of these analyses, and how is the inferred ancestral order determined?

These analyses did not involve any gene order. Indeed, the variant calling was done by mapping the draft assemblies on a reference genome for each species (Lp: Paris strain, Ab: AB5075 strain). The estimation of genetic linkage used the frequencies of the previously called biallelic SNPs that were polymorphic in transformable and non-transformable strains.

3.26 Lines 715-737: Presumably all the interesting patterns breakdown when data are permuted or partially randomized?

When data are partially randomized, the phylogenetic signal values are modified as it is shown in Figure S17, but not dramatically so (high Spearman coefficients of correlation). This is because many recombination tracts are smaller than the size of genes and the others are only partially affected by the permutation.

3.27 Lines 739-824: Nice detailed treatment. Given all this extra annotation, it would be extremely useful if a supplement were included that provided MGE "burden" for each strain.

We added a supplement table summing up the amount of MGEs each strain carries (Table S10).

3.28 Lines 826-877: Great!

Thank you!

### Reviewer #4:

We thank the reviewer for the positive assessment of our work and the relevant comments and criticisms.

Assessment:

In this manuscript, the authors describe variation in competence in two model species - Acinetobacter baumannii and Legionella pneumophila - and carry out extensive bioinformatics analysis to identify genetics determinants of this observed variation. This is a comprehensive study that has the potential to make an important conceptual advance to our understanding of the driving forces behind the evolution of natural competence. As a non-bioinformatics specialist, I will focus my review on the conceptual advances rather than the methodological underpinnings.

Comments :

Major:

4.1- The large scale transformation assays in Fig. 1 are well-designed and produce exciting data that feed into the majority of the downstream analysis. Developing and optimising this high-throughput assay is extremely valuable, and reveals high levels of between strain variation in recombination when supplying DNA encoding luciferase. My main concern with this assay is that it is unclear how sensitive the results are to variation in growth conditions. For example, different strains may have evolved to switch competence on or off under different environmental conditions. If so, this could result in (many) false-negative results in the assay, with (potentially major) implications for the downstream modelling of competence gain/loss and GWAS analysis. In addition, recombination success/failure may depend on micro-homology between the luciferase DNA and the bacterial chromosome, which may not follow the phylogeny if homology is within mobile elements, or if lack of recombination
is due to sequence-specific nucleases in the bacteria that move by HGT. These factors have not been sufficiently taken into account. Perhaps, the authors could carry out assays on a selected (small) number of recombination +ve and -ve isolates to examine the robustness of the Fig. 1 data to variation in growth conditions (e.g. varying nutrients, temperature, growth phase) and DNA sequence (e.g. with/without homology arms)? 

Part of this is addressed by the response to reviewer #3. Please see answers in 3.1, 3.2.

With respect to variation in conditions to induce competence we can distinguish Ab and Lp. For Lp, there are no other conditions to be tested. Lp grows only in the tested medium (AYE) and becomes competent at 30°C. For Ab, too little is known about the conditions that induce competence to test variations in the conditions. Of course, we cannot rule out the possibility that some strains have evolved to express competence under other conditions, but this could have been achieved by variations in the regulation system.

4.2- Transformation rates evolve as a jump process - I felt this section was rather dense and quite inaccessible to general readership of PLoS Biol. I suggest the authors to rework this part of the paper , explaining a more detailed description of the question , approach, explanation of how these models work, confidence in the models and ability to discriminate between them. Perhaps including control genes / traits for which patterns of evolution are well established would be helpful to illustrate differences in dynamics (e.g. housekeeping genes?).

This section was re-written. See answer to comment 3.5.

4.3- The GWAS data point towards MGE gene candidates that inhibit competence / transformation, as well as genes that are associated with increased competence. The obvious next step will be to validate at least some of the most promising candidates by deleting them from non-competent strains and/or introducing them into competent strains, and demonstrate their effect on recombination using the assay in Fig. 1. Including such experimental validations would really make the study shine, and alleviate any concerns to do with experimental biases and downstream modelling referred to in comment 1.

We agree with the reviewer that experimental validation of GWAS candidates is key. Our most promising candidate in Lp, the sncRNA rocRp, was already experimentally validated (Durieux et al, PNAS, 2019). This supports our GWAS findings. For Ab, it would have also been nice to have some validation of the phages inhibitory role against transformation but it raises several challenges. The main one being the identification of the specific phage(s) responsible for non-transformability among the several phages the strain carries and its/their removal. This is ongoing, but it is proving harder than expected and will be the subject of a subsequent publication.

Minor:

4.5 L132 The authors state that "transformation assays were performed multiple times and revealed good concordance" - it would be good to provide a more quantitative description of the variation observed, and to make these data available in SI.

See answer to comment 3.22.

4.6 L199 Please indicate effect size for both species

The effect size is now indicated for both species.

4.7 L222  Delta-R^2 statistic - Please provide calculation for each species independently

The difference in r^2^ averaged along the genome was provided for each species.

4.8 L249-252 Is this analysis done using a phylogenetically-aware model?

This analysis was done by simply performing a chi2 test of association between the transformation phenotype or the source of the strain and the presence of an interrupted *comM*. We could have taken into account the phylogeny by using a phylogenetic logistic regression model with phyloglm. When doing so, we obtain: TF_binary~pseudogenized comM: effect=-1.50, p=9.447e-09. Source ~pseudogenized comM with clinical=0 and environmental=1: effect= -1.54014, p=6.467e-08.

**Action**: These results are now added in the main text and in Table S11.

4.9 L308 - Please provide statistics to support claim that majority of inhibitors are MGE encoded

See answer to comment 2.5 that also highlighted this point.
