# Supplementary material for: Intragenomic conflicts with plasmids and chromosomal mobile genetic elements drive the evolution of natural transformation within species
Source: PLoS Biol. 2024 Oct 14;22(10):e3002814. doi: 10.1371/journal.pbio.3002814 (PMC11472951; doi:10.1371/journal.pbio.3002814)
Supplement: Attachment — Submitted filename: reviewer_comments_v3.docx [file pbio.3002814.s049.docx]

Reviewer #1:

I thank the authors for the time they have taken in addressing to my comments; their response is excellent. I have two remaining major comments, and some minor comments.

We thank the reviewer for the comments.

Major comments :

1.1.a. I thank the authors for referencing the manuscript by Vesel et al, which provides an alternative perspective on a similar analysis. It would be helpful if the authors could comment on whether the R-M system unitigs that are associated with the differences in transformation (Table S6) correspond to the R-M systems that were identified and characterised in Vesel et al. It would be interesting to test whether DNA from different sources affected the efficiency of transformation of a bacterium harboring one of these R-M systems, but I sympathise with the view that such experiments are beyond the scope of this manuscript, which already contains extensive work.

Vesel et al described a system specific to A118 strain (a Type 2 system that uses methylation as a marker of self). This system is in a region of high genomic variability and our experience is that such systems are often strain-specific. This is confirmed in the original article, which explains why it can’t be identified by the GWAS.

We extracted the unitigs that mapped to the restriction modification systems we had detected. We then performed a nucleotide alignment of these unitigs to the coding sequences composing the Vesel et al. RM system, that is to say H0N27 10820 (methylase), H0N27 10825 (helix-turn helix transcriptional regulator) and H0N27 10830 (restriction endonuclease), with *blast*. There was no match between these unitigs and Vesel et al’s RM system components. Hence, we identify other RM-systems. We do not exclude that the RM systems pointed out by the unitigs may have a similar mode of action of Vesel et al’s RM system.

1.1.b. The authors state in their response that "As expected, both were significatively and negatively associated with transformation but phages in Ab and conjugative systems in Lp had a stronger inhibitory effect than restriction modification systems (Ab: Phages: effect=-0.97, p=4.3x10-3; RM: effect=-0.61, p=3.55x10-8 and Lp: Conjugative systems: effect=-0.76, p=1.4x10-4; RM: effect=-0.41, p=1.09x10-9)."

In both species, although the point estimate of the inhibitory effect of mobile elements is of a larger magnitude than that of R-M systems in these paired univariate analyses, the effect of R-M systems is more significant. Furthermore, no confidence intervals are provided for these estimates of the inhibitory effects, to determine whether they are significantly different from one another. Finally, there are biological reasons to expect that the distribution of mobile elements and R-M systems would not be independent of one another. Therefore it would be helpful to conduct a multivariate analysis of the effects of R-M systems and mobile elements, to quantify the relative contributions of the mobile element and R-M loci (including confidence intervals).

Phyloglm is a multivariate analysis but here we only tested two variables which are the number of phages in Ab, conjugative systems in Lp on one hand and the restriction modification systems on the other hand. It is important to remember that the more we include variables in a multivariate analysis, the more there is a loss of power of the analysis and thus a loss of significancy but also a risk of overfitting the data. However, we agree it is necessary to get the confidence interval to be able to properly compare the effect of MGE and RM system. We computed confidence intervals (alpha=0.05) from the estimates of the stderr obtained given by phyloglm.

Lp: Effect of conjugative systems=-0.75663 [-0.779;-0.735] while effect of RM=-0.41965 [-0.441;-0.398]

Ab: Effect of phages: -0.97168 [-1.038;-0.905] while effect of RM=-0.61175 [-0.634;-0.590].

This suggests that MGEs have a stronger effect than RM systems to the transformation phenotype.

The confidence intervals were added to the results.

1.2. The authors' analysis of plasmid diversity in their response is very interesting. However, such clarity is missing from the updated manuscript, which contains the confusing passage: "By blocking transformation, RocRp could also contribute to the preservation of chromosomal MGEs in positive epistatic interaction with the plasmid. In addition, plasmids could encode RocRp to avoid recombination with DNA entering by transformation from other partly homologous plasmids that could lead to deletion of non-homologous regions and/or create plasmid instability. Our results show that similar plasmids lacking RocRp do exist (Figure S11). In any case, these results strongly suggest that intragenomic conflicts between MGEs and natural transformation are not restricted to the effect of chromosome curing.".

I think it is worthwhile making it explicit that the rocRp gene is not widespread across multiple plasmids, but instead confined to a cluster of highly-related plasmids, given the RNA was described in Durieux et al, which had the title "Diverse conjugative elements silence natural transformation in Legionella species". A comparison of pLPL and pFFI102 suggests the RNA is within a cluster of genes that function to preserve the mobile element (e.g. an anti-restriction protein, a toxin-antitoxin system), and an integrase (plpl0043). As rocRp is shared with ICEs in other Legionella species (where chromosomal curing is a valid reason for carrying the RNA), it may be that this is a gene cluster for preserving conjugative elements that has been inserted once into the ancestor of the plasmids that carry rocRp through site-specific recombination. As it has only entered the L. pneumophila population once, it might be that rocRp actually has no direct role in benefitting the plasmid,

but instead may be hitchiking on function of the other genes that do provide an advantage to the replicon (e.g. that encoding the anti-restriction protein). Furthermore, it may be that rocRp prevents homologous recombination between related plasmids that may delete the anti-restriction gene. However, given the absence of multiple parallel acquisitions of the rocRp gene by different plasmids, there is currently no direct evidence of rocRp itself directly benefitting the plamids, and therefore chromosomal curing may remain a valid explanation for its evolution. Nevertheless, it is certainly important to think about the possibilities for fresh ideas.

We have analysed the similarity between RocRp plasmids in complete genomes (a much smaller dataset but one for which we can be sure of the integrity of the elements) and while they were all related, they make three groups with weighted gene repertoire relatedness < 90%. We find the hypothesis proposed above unlikely. It would require presence of a gene that is proposed of being of no use for plasmids in at least three different (even if related) groups of plasmids that are present in many strains. It assumes such a gene is conserved in the plasmid for a long-time scale even though the gene is deleterious to the host (or that there were three independent acquisitions of RocRp, which seems just as unlikely for a trait that is deleterious to the host and neutral to the plasmid). Given that we have shown the blocking of transformation by RocRp (previous work), that its association is very strong in the GWAS, that it is never found in the chromosome in the species, and that transformation loss is deleterious to the bacteria, we find the coincidence hypothesis not parsimonious, and more evidence is necessary before putting it forward. We have modified the manuscript to clarify the data on the similarity between plasmids.

Minor comments :

1.3. The Discussion now includes the passage, "Transformation itself may be costly under certain circumstances and favour for a short-term bacteria that lost it. But in the long term, the inability to take advantage of transformation may result in the purge of these lineages by natural selection." The authors themselves highlight this as potentially requiring editing for clarity, which I think would improve the work.

We modified the passage in the discussion.

1.4. Table S6a contains the phrase "conjugal protein", which should be "conjugative protein"

This phrasing corresponds to the annotation given by prokka and eggnog and refers to conjugal transfer protein. No modification was made.

Reviewer #2:

I thank the authors for a much clearer second version of their manuscript, which addressed many points raised by 4 reviewers.

We thank the reviewer for the comments.

2.1. I concede to the authors that even though gubbins is not appropriate in the general case, it might be "less wrong" in the context of this work. The authors should perhaps mention this caveat (and perhaps a call to the community to implement an appropriate substitute method) in the Discussion and not just in the Methods.

We thank the reviewer for the suggestion. We would like to reiterate that even though Gubbins has its caveats, it is a standard approach and accurate when one focuses on very recent events of recombination. The limitations of Gubbins are discussed in the Methods and now briefly mentioned in the discussion. We agree that a community effort to improve recombination detection is important, but we think a community call would be more pertinent in another context.

2.2. While I appreciate the clarification regarding the horizontal lines in Figure 4A, I remain unconvinced that the authors are selecting an appropriate p-value threshold in their GWAS, even more so now that I can see a QQ-plot. For Acinetobacter in particular, the authors observe a flat line in the QQ-plot, and -log10 of the p-values that barely go over 6. This would lead me to expect no significant hits or just a handful of spurious ones; instead the authors report 1262 genes associated with increased or decreased transformability. That is ~35% of all genes, which is hard to believe and most certainly an artifact; unless the authors have a good explanation of why one third of the genome modulates this one phenotype. The authors should look again at their QQ plot and apply a more stringent threshold to come to a more realistic set of associated genes (if there are any). Applying a Bonferroni correction on the number of unique tested patterns (note that it is significantly less

than the number of tested unitigs) is a recognized good practice which would provide a more sober result. I don't expect this to change much the results for Legionella, which has a few genes with very small p-values.

We respectfully disagree.

- We would like to correct the reviewer’s statement: the “1262 genes associated with increased or decreased transformability” in Ab highlighted here, are actually 1262 pangenome families found among the 31103 pangenome families of the pangenome collection, which thus represent less than 5% of the pangenome that could explain the non-transformable phenotype. Given the linkage between the genes in MGEs (which necessarily inflates the numbers) this is not so “hard to believe”.
- We agree that by looking at the QQ-plots it is obvious that associations in Ab are not as significant as in Lp (as stated in our text). The main reason is that in Lp almost a third of the genomes have the inhibitory element which gives an extremely good power for the analysis. In Ab the situation is not as ideal as in Lp: there are many putative transformation-inhibiting elements sharing homologs. Nonetheless, the Ab QQ-plot still shows a difference between the observed p-value and the expected p-value at the highest p-values that is inconsistent with the results of the Bonferroni correction (see below).
- We do not deny that applying Bonferroni correction on the number of unique tested patterns is a recognized good practice. Yet the use of Benjamini-Hochberg (BH) is also a recognized good practice. The choice of one or the other method depends on what we want to achieve. If Bonferroni controls in a very stringent manner the probability of having a false positive, BH helps increasing the power of the analysis by controlling instead the proportion of false positives. Despite the possibility of increasing false positives due to the use of BH threshold, the rest of the analyses, especially the phyloglm analysis, also supports the set of pangenome families that we found.
- We would like to emphasize that contrary to many GWAS, our focus is on genes matched by the unitigs, not on the unitigs themselves. This makes the Bonferroni correction on the number of unique unitigs even more conservative.
- Nevertheless, we applied a Bonferroni correction on the number of unique tested patterns (Ab:357728, Lp:406151) and obtained the Bonferroni significancy threshold in both species (Ab:1.4x10^-7^, Lp:1.23x10^-7^). According to this threshold, 65 gene families are negatively and 6 positively associated with transformation in Lp. In Ab, only one gene family carried by prophage is associated with the phenotype and in a negative manner. It is important to note that the one gene (ComM) shown to affect recombination experimentally in Ab is lost when using the Bonferroni correction, but identified correctly with FDR. Similarly, R-M systems shown to be modulators of transformation in several species are all lost with the Bonferroni correction but identified with the FDR.

Following the reviewer comment, and to provide all the information to the reader, the numbers associated with Bonferroni correction were added to the manuscript.

Reviewer #3:

We thank the reviewer for the clarification of the previous comments.

Thanks to the authors for the thorough response to reviewers. Most comments from all four of us were adequately addressed. I would like to clarify two points I made that the authors still didn't quite address:

3.1 Chicken-and-egg: In 3.6, the authors misunderstand my issue: Although MGE accumulation may slowly make strains less fit, this was not what I meant. Instead, the model itself presents two ways in which MGE-transformation can be in conflict that are difficult to resolve: (a) MGEs may mutate competence genes and make strains less transformable, in order to keep from being cured. This is the favored model of the authors, and certainly the odd comM "targeting" and RocRp RNAs are possible examples of this. However, significantly more broadly: (b) a simple point mutation in a competence gene that reduces transformability may lead to the accumulation of MGEs, irrespective of any direct effect of the MGEs themselves, since MGE "curing" role by transformation would be reduced. Indeed, there's a large body of experimental and theoretical work in higher eukaryotes that demonstrates how areas with reduced recombination (e.g. near centromeres, etc) accumulate MGEs, simply by virtue of

having lower recombination rates. I think that the model the authors' propose is a bit wedded to the idea of certain MGEs directly impacting transformation, rather than the simply converse that they cannot be cured in non-transformable strains. Thus, many of the associations seen in their GWAS with MGEs (especially since these are abundant but weak) are merely associations and not causal. A role for MGE accumulation as a Muller's ratchet leading to extinction of low/non-transformable strains might indeed explain a lot about the distribution of competence within and among species. My point is that many of the MGE associations seen do not in any way suggest these are all affecting transformation rates.

We thank the reviewer for the suggestion. We agree that it is possible that some of the MGE associations found in the GWAS are not affecting the transformation rates directly but exist because of the larger number of MGEs in non-transformable strains. We added a sentence to acknowledge the existence of such MGE associations.

3.2. Luminescence correlations with transformation rates: The authors somewhat address me and Reviewer #4's concerns, but the dilution experiment only says that the technical method is reasonable and the use of known non-transformable isolates for baseline correction does not address the main issues we raised about natural genetic variation in competence induction, expression of the luc marker, or growth. This would require a handful of genetically distinct isolates with luminescence values across the range of measured values to actually have transformation experiments conducted. The authors did further elaborate why they think their values are correlated to transformation rates, but they should acknowledge that there's probably a fiar amount of "slop" in the values.

Variation in competence induction or growth:

We certainly cannot exclude variations in competence induction, growth and variation in the kinetic of competence induction. In Ab, competence is expressed in exponential phase (Vesel, J. Bact 2021), while in Lp it occurs at the transition between exponential and stationary (Attaiech, PNAS 2016). It is possible that some strains display different kinetic of expression. To limit this effect, transformation experiments were conducted with transforming DNA present during the entire growth experiment. Observed variation in natural transformation are likely not due to variation in competence induction.

Expression of the luc marker:

The nanoluc marker was designed to limit variation in its expression. Nluc is placed under a synthetic, strong constitutive promoter. It is still possible that some variations are present between isolates, but these are unlikely to extend over several order of magnitude. To further test this possibility, we transformed a random set of 60 isolates with the nanoluc marker (which also confers resistance to kanamycin). Because not all isolates are kanamycin-sensitive, we ended up with 15 transformed isolates. We cultured them and measured the luminescence level. These strains show 100-fold variations in transformation frequencies determined by nanoluc, yet quantification of the amount of nanoluc they produce shows only a 3-fold difference between the minimum and maximum values. Thus, variations in transformation frequencies could not be attributed to variations in expression of nanoluc between strains.

We added the evaluation of expression of the luc marker in Material and Methods and in Table S11.

Reviewer #4:

All my comments have been addressed in a satisfactory manner.
